# Red fluorescent CEPIA indicators for visualization of Ca^2+^ dynamics in mitochondria

**DOI:** 10.1038/s41598-020-59707-8

**Published:** 2020-02-18

**Authors:** Kazunori Kanemaru, Junji Suzuki, Isamu Taiko, Masamitsu Iino

**Affiliations:** 1 Division of Cellular and Molecular Pharmacology, Nihon University School of Medicine, Tokyo, 173-8610 Japan; 2 Department of Physiology, University of California San Francisco, San Francisco, CA 94158 USA; 3 Department of Physiology, Nihon University School of Medicine, Tokyo, 173-8610 Japan

**Keywords:** Biological techniques, Biotechnology, Cell biology

## Abstract

Mitochondrial Ca^2+^ dynamics are involved in the regulation of multifarious cellular processes, including intracellular Ca^2+^ signalling, cell metabolism and cell death. Use of mitochondria-targeted genetically encoded Ca^2+^ indicators has revealed intercellular and subcellular heterogeneity of mitochondrial Ca^2+^ dynamics, which are assumed to be determined by distinct thresholds of Ca^2+^ increases at each subcellular mitochondrial domain. The balance between Ca^2+^ influx through the mitochondrial calcium uniporter and extrusion by cation exchangers across the inner mitochondrial membrane may define the threshold; however, the precise mechanisms remain to be further explored. We here report the new red fluorescent genetically encoded Ca^2+^ indicators, R-CEPIA3*mt* and R-CEPIA4*mt*, which are targeted to mitochondria and their Ca^2+^ affinities are engineered to match the intramitochondrial Ca^2+^ concentrations. They enable visualization of mitochondrial Ca^2+^ dynamics with high spatiotemporal resolution in parallel with the use of green fluorescent probes and optogenetic tools. Thus, R-CEPIA3*mt* and R-CEPIA4*mt* are expected to be a useful tool for elucidating the mechanisms of the complex mitochondrial Ca^2+^ dynamics and their functions.

## Introduction

Mitochondrial Ca^2+^ dynamics contribute to the control of various cellular functions such as formation of spatiotemporal patterns of cytosolic Ca^2+^ dynamics, cellular metabolism and cell survival^1^. Ca^2+^ concentrations in the mitochondrial matrix are regulated by the balance between the influx of Ca^2+^ through the mitochondrial Ca^2+^ uniporter (MCU) and the efflux of Ca^2+^ by Na^+^/Ca^2+^ or H^+^/Ca^2+^ exchangers^1–3^. Recent studies have elucidated the molecular identity of the channel and regulatory components of MCU^4–11^ as well as Na^+^-Ca^2+^-Li^+^ exchanger in the mitochondrial inner membrane^12,13^. Furthermore, the advent of genetically encoded Ca^2+^ indicators (GECIs) that are targeted to the mitochondrial matrix has enabled monitoring mitochondrial Ca^2+^ dynamics with high spatiotemporal resolution, revealing both the subcellular and intercellular heterogeneity of mitochondrial Ca^2+^ responses upon agonist-induced increases in the cytosolic Ca^2+^ concentration^14–16^. These imaging results suggest that the threshold of the net Ca^2+^ flux into the mitochondrial matrix is differentially determined in individual cells or even in each subcellular mitochondrial domain. However, the mechanism of these heterogeneous mitochondrial Ca^2+^ dynamics and their functional significance remains to be clarified. Thus, further analyses combined with high-resolution mitochondrial Ca^2+^ imaging are required.

We have previously developed a Ca^2+^ indicator protein family of Calcium-measuring organelle-Entrapped Protein IndicAtors (CEPIA) to visualize Ca^2+^ signals in both the endoplasmic reticulum (ER) and mitochondria^15^. ER-targeted CEPIAs have *K*_d_ values for Ca^2+^ ranging between 558 and 672 µM, which are higher than those of other ER-targeted GECIs such as D1ER^17^, GCaMPer^18^, ER-GCaMPs^19^, and ER-LAR-GECOs^20^. Mitochondrial Ca^2+^ imaging analyses using green fluorescent protein (GFP)-based CEPIA variants with lower Ca^2+^ affinities (*K*_d_ = 14.5 or 90.2 µM) than cytosolic Ca^2+^ indicators suggested that mitochondrial Ca^2+^ concentrations can increase beyond 50 µM in a small fraction of HeLa cells. While a red fluorescent protein (RFP)-based low-affinity GECI, mito-LAR-GECO1.2 (*K*_d_ = 12 µM)^20^, has been developed, lower-affinity mitochondrial RFP-based CEPIAs have not been developed, yet. Moreover, GFP-based GECIs cannot be used simultaneously with other green fluorescent imaging tools, including synthetic Ca^2+^ indicators, nor are they simultaneously used with optogenetic tools that are activated by blue light, such as channelrhodopsin-2 and OptoXRs^21,22^. These limitations can be circumvented by GECIs with longer excitation and emission wave lengths. Therefore, CEPIA variants with red fluorescence may allow simultaneous use of other optical tools to increase the utility of mitochondria-targeted GECIs.

To this end, we generated the red-fluorescent CEPIA*mt* variants, R-CEPIA3*mt* and RCEPIA4*mt*, of which the Ca^2+^-affinity was optimized to measure mitochondrial Ca^2+^ concentrations. These variants allow visualization of mitochondrial Ca^2+^ signals with high spatiotemporal resolution that enables the detection of mitochondrial Ca^2+^ dynamics at subcellular local domains. Furthermore, simultaneous use of green fluorescent Ca^2+^ indicators and optogenetic tools is possible. Thus, R-CEPIA3*mt* and R-CEPIA4*mt* are expected to be a valuable tool for obtaining deeper insight into the cellular functions of mitochondrial Ca^2+^ dynamics.

## Results

### *In vitro* characterization of R-CEPIA3*mt* and R-CEPIA4*mt*

**Figure 1:**
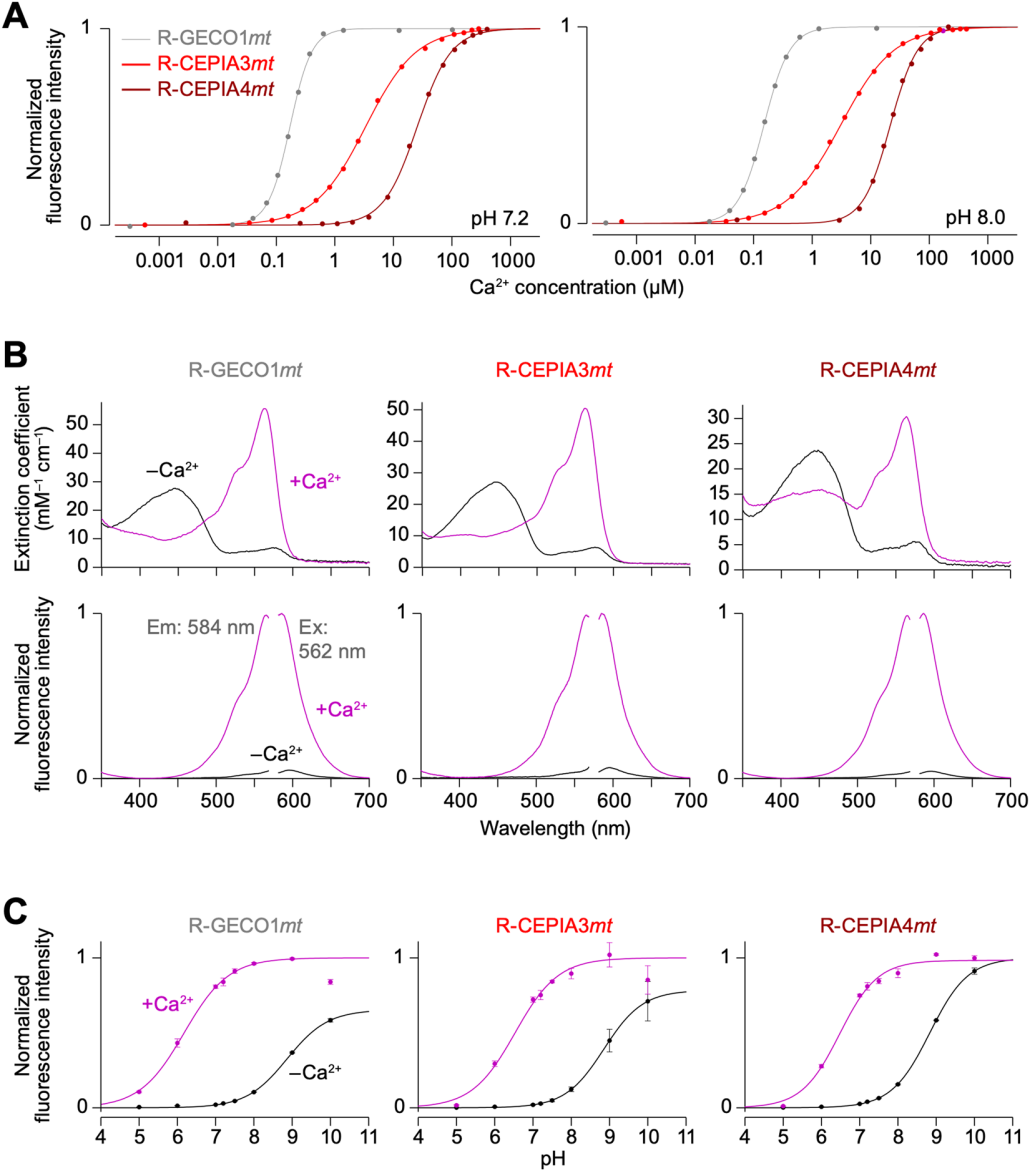
*In vitro* characterization of R-CEPIA3*mt* and R-CEPIA4*mt*. All the extracted parameters are summarized in Table 1. (**A**) *In vitro* Ca^2+^ titration curves of R-GECO1*mt* (gray), R-CEPIA3*mt* (red) and R-CEPIA4*mt* (brown) at pH 7.2 (left) or 8.0 (right) solution. (**B**) Absorption (upper), excitation and emission (lower) spectra of R-GECO1*mt* (left), R-CEPIA3*mt* (middle) and R-CEPIA4*mt* (right) in Ca^2+^-containing (1 mM, magenta) or Ca^2+^-free (1 mM EGTA, black) solution. (**C**) pH titration curves of R-GECO1*mt* (left), R-CEPIA3*mt* (middle) and R-CEPIA4*mt* (right) in Ca^2+^-containing (1 mM, magenta) or Ca^2+^-free (1 mM EGTA, black) solution. The plots were fitted by a single Hill equation. Mean ± SEM (*n* = 3).

On the basis of an amino acid substitution strategy to produce low Ca^2+^ affinity variants of CEPIA^15^, we generated R-CEPIA3*mt* and R-CEPIA4*mt* by modifying one (E31D) and three (E31D, F92W and D133E) amino acids, respectively, in the calmodulin domain of R-GECO1*mt* (Supplementary Fig. 1A). As expected, these mutant indicators had reduced Ca^2+^ affinities (*K*_d_ = 3.7 µM for R-CEPIA3*mt*; *K*_d_ = 26.9 µM for R-CEPIA4*mt*) and high dynamic ranges (Fig. 1A and Table 1) without apparent alterations in the extinction coefficient, both excitation and emission spectra, and pH dependence of the original R-GECO1*mt* (Fig. 1B,C and Table 1). Notably, R-CEPIA3*mt* had a broader dynamic range with reduced cooperativity compared with those of R-GECO1*mt* and R-CEPIA4*mt*, indicating that R-CEPIA3*mt* may be useful for detecting dynamic changes in mitochondrial Ca^2+^ levels ranging from 0.5 to 100 µM.

**Table 1:** Properties of R-CEPIA3*mt* and R-CEPIA4*mt*.

Probe	Ca^2+^	ε (mM^−1^ cm^−1^) (λ_ABS_*)	p*K*_a_^†^	λ_Ex_^*^	pH 7.2			pH 8.0		
					*K*_d_ for Ca^2+^ (μM)	Dynamic range^‡^	Hill coefficient	*K*_d_ for Ca^2+^ (μM)	Dynamic range^‡^	Hill coefficient
R-GECO1*mt*	−	27 (445), 7 (576)	8.9	565	0.19 ± 0.02^§^	22.2 ± 0.5^§^	2.20 ± 0.10^§^	0.14 ± 0.01^§^	8.8 ± 0.1^§^	2.12 ± 0.10^§^
	+	54 (562)	6.2							
R-CEPIA3*mt*	−	26 (445), 6 (576)	8.9	565	3.7 ± 0.5	30.0 ± 1.5	0.96 ± 0.02	3.3 ± 0.2	8.9 ± 0.1	0.93 ± 0.01
	+	49 (562)	6.5							
R-CEPIA4*mt*	−	25 (445), 6 (576)	8.8	565	26.9 ± 1.0	23.9 ± 0.4	1.51 ± 0.01	21.4 ± 0.3	4.7 ± 0.1	1.86 ± 0.02
	+	17 (450), 30 (562)	6.5							

*λ_ABS_ and λ_Ex_ are the maximum wavelength of absorption and fluorescence excitation spectra, respectively.

^†^p*K*a is determined as the pH at half-maximal fluorescence intensity calculated by fitting Hill equation to each plot.

^‡^Dynamic range indicates the ratio of the maximum to minimum fluorescence intensity (*F*_max_/*F*_min_).

^§^Mean ± s.e.m.

### Mitochondrial Ca^2+^ signals visualized by R-CEPIA3*mt* and R-CEPIA4*mt*

**Figure 2:**
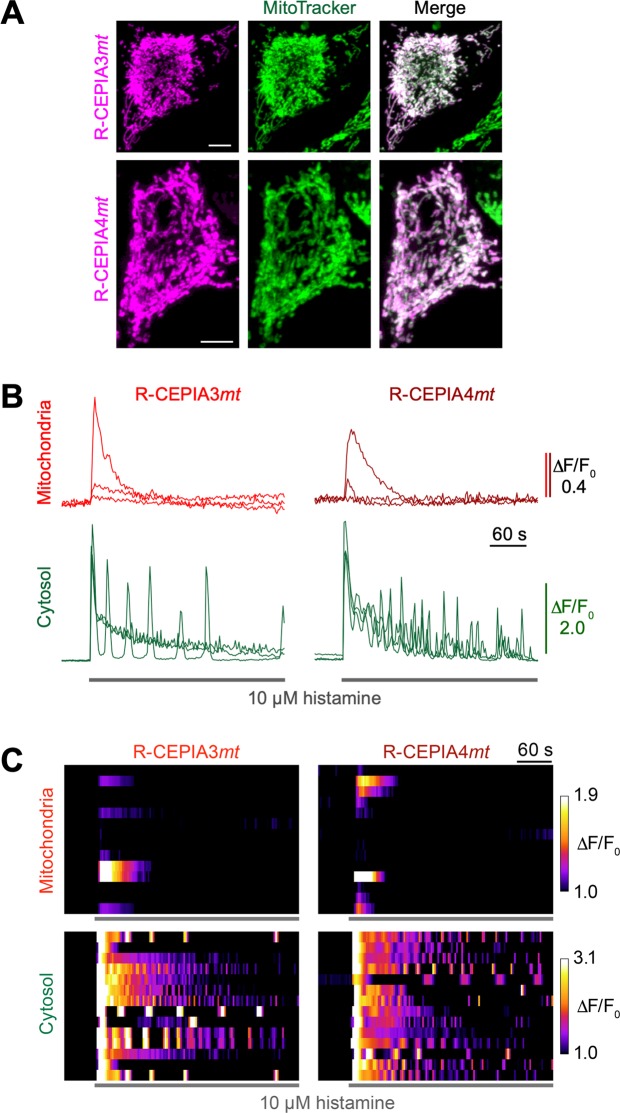
Global mitochondrial Ca^2+^ signals visualized by R-CEPIA3*mt* and R-CEPIA4*mt*. (**A**) Representative images of HeLa cells expressing R-CEPIA3*mt* (upper) or R-CEPIA4*mt* (lower). Fluorescence of R-CEPIAs (left), MitoTracker Green staining (middle) and the merged images (right) are shown. Scale bars, 5 µm. (**B**) Time course of agonist-induced Ca^2+^ response in the mitochondria (upper) and cytosol (lower) in three representative HeLa cells expressing R-CEPIA3*mt* (left) or R-CEPIA4*mt* (right). Cytosolic Ca^2+^ signals were visualized by a green fluorescent synthetic Ca^2+^ indicator, Cal-520. (**C)** Heat maps of cell population data (n = 14) of global Ca^2+^ signals in mitochondria (upper) and cytosol (lower). Each horizontal strip corresponds to the time course of the Ca^2+^ signal in each cell.

Using these CEPIA variants, we visualized mitochondrial Ca^2+^ signals in HeLa cells. The mitochondrial distribution of both variants was confirmed by colocalization with MitoTracker Green (Fig. 2A). Simultaneous Ca^2+^ imaging in mitochondria and the cytosol (the latter was visualized by the green fluorescent Ca^2+^ indicator, Cal-520) demonstrated that only a fraction of the cells (35.7%, *n* = 34 for R-CEPIA3*mt*; 28.6%, *n* = 38 for R-CEPIA4*mt*) showed a transient mitochondrial Ca^2+^ increase in response to cytosolic Ca^2+^ elevations induced by the inositol trisphosphate-producing agonist, histamine (Fig. 2B,C). Interestingly, mitochondrial Ca^2+^ transients were elicited only by the initial peak of the cytosolic Ca^2+^ oscillations, which reached the threshold of mitochondrial Ca^2+^ increases. Similar observations have previously been reported using GFP-based mitochondrial CEPIAs^15^.

**Figure 3:**
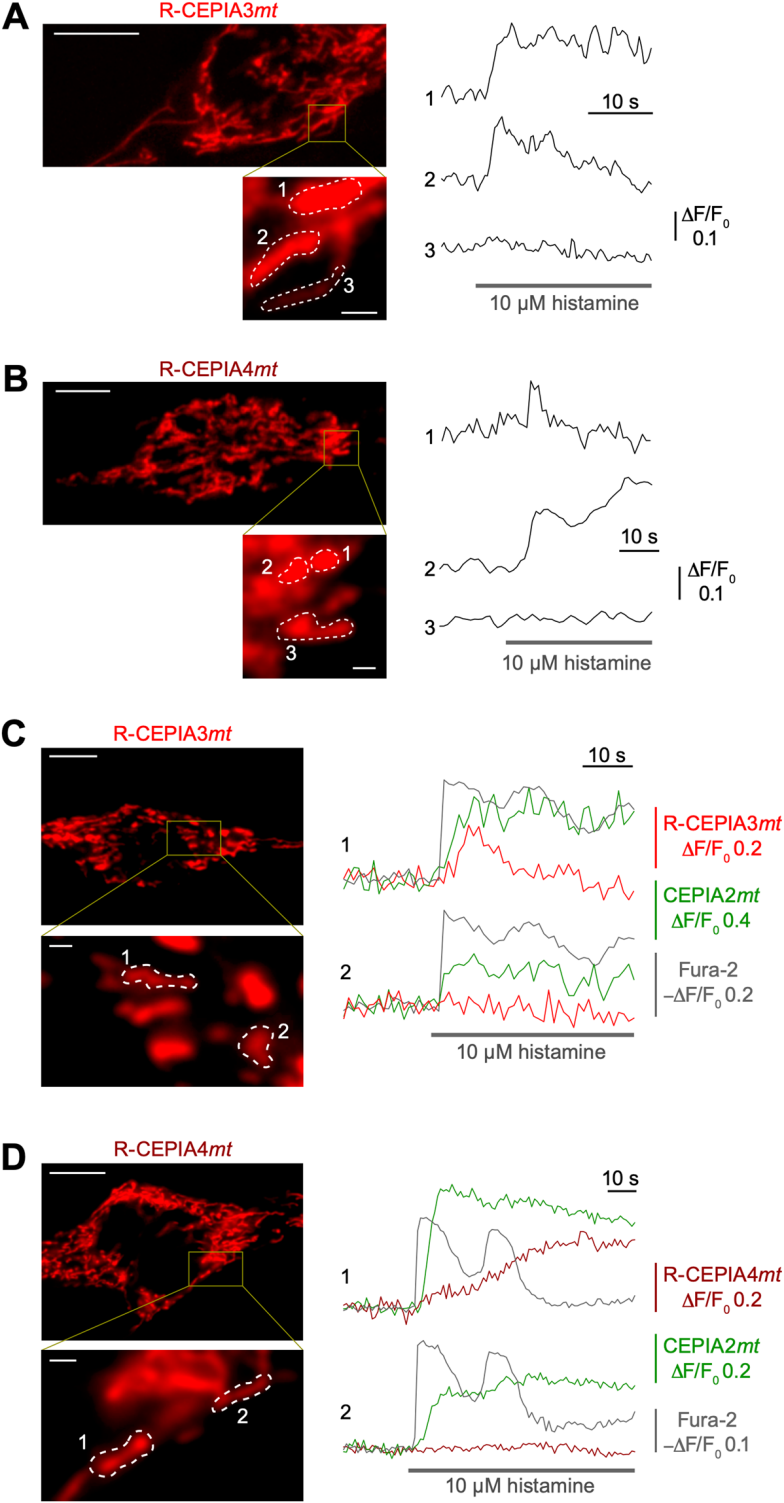
Local mitochondrial Ca^2+^ signals visualized by R-CEPIA3*mt* and R-CEPIA4*mt*. **(A**,**B**) Representative red fluorescence images and time courses of agonist-induced Ca^2+^ signals in subcellular mitochondrial domains in a HeLa cell expressing R-CEPIA3*mt* (**A**) or R-CEPIA4*mt* (**B**). Regions of interest (ROI) are indicated in high magnification images shown in the left lower panels. Scale bars, 10 µm (upper) and 1 µm (lower). (**C**,**D**) Simultaneous Ca^2+^ imaging of mitochondria with low Ca^2+^ affinity R-CEPIAs (red fluorescence), high Ca^2+^ affinity CEPIA2*mt* (green fluorescence) and the cytosolic Ca^2+^ indicator, fura-2 (405-nm excitation). Representative red fluorescence images and time courses of agonist-induced Ca^2+^ signals in subcellular mitochondrial domains in a HeLa cell are shown. ROI are indicated in high magnification images shown in the left lower panels. The cells expressing both R-CEPIA3*mt* and CEPIA2*mt* (**C**), and both R-CEPIA4*mt* and CEPIA2*mt* (**D**) were loaded with fura-2. Scale bars, 10 µm (upper) and 1 µm (lower).

To examine the spatial resolution of R-CEPIA3*mt* and R-CEPIA4*mt*, we next focused on subcellular mitochondrial domains in single HeLa cells. Both R-CEPIA*mt* variants successfully detected heterogenous Ca^2+^ signals in mitochondrial subdomains within close proximity (Fig. 3A,B). We next performed simultaneous imaging of mitochondrial Ca^2+^ using two CEPIA*mt* variants with different colours and Ca^2+^ affinities to effectively expand the range of Ca^2+^ concentrations detectable with the indicators. HeLa cells were co-transfected with R-CEPIA3*mt* or R-CEPIA4*mt* and the GFP-based high Ca^2+^ affinity CEPIA2*mt* (*K*_d_ = 160 nM)^15^. Additionally, the cells were loaded with fura-2 for simultaneous detection of cytosolic Ca^2+^ dynamics. As shown in Fig. 3C,D, we found that individual HeLa cells had heterogenous mitochondrial domains, of which Ca^2+^ signals were detected either by both CEPIA2*mt* and one of the R-CEPIA*mt* variants (domain 1 in Fig. 3C,D) or only by CEPIA2*mt* (domain 2 in Fig. 3C,D). It is remarkable that agonist-induced mitochondrial Ca^2+^ dynamics were detected by indicators with varying affinities to Ca^2+^. On the basis of the calibrations of the indicators (Fig. 1A), these results suggest that each mitochondrial subdomain reaches distinct Ca^2+^ levels that range between 0.1 and 100 µM.

### pH dependence of R-CEPIA3*mt* and R-CEPIA4*mt*

Using HeLa cells co-expressing CEPIA2*mt* and one of the new R-CEPIA*mt* variants, we examined the pH dependence of R-CEPIA3*mt* and R-CEPIA4*mt*. Although alkalization by NH_4_Cl induced a sustained elevation in the fluorescence intensity of all mitochondrial CEPIAs, the amplitude of the change in R-CEPIA3*mt* and R-CEPIA4*mt* was significantly smaller than that of CEPIA2*mt* (Supplementary Fig. 2). This is attributable to the acidity shifted acid dissociation constant of R-CEPIA3*mt* and R-CEPIA4*mt* (Table 1; p*K*_a_ = 6.5 in the presence of Ca^2+^ for both the R-CEPIA*mt* variants) compared with the GFP-based CEPIAs (p*K*_a_ = 7.0 in the presence of Ca^2+^ for CEPIA2*mt*, Supplementary Fig. 1B)^15^. Thus, R-CEPIA3*mt* and R-CEPIA4*mt* enable a more stable detection of Ca^2+^ signals in mitochondria, which may undergo dynamic pH changes.

### Simultaneous use of an optogenetic tool and mitochondrial Ca^2+^ indicators

**Figure 4:**
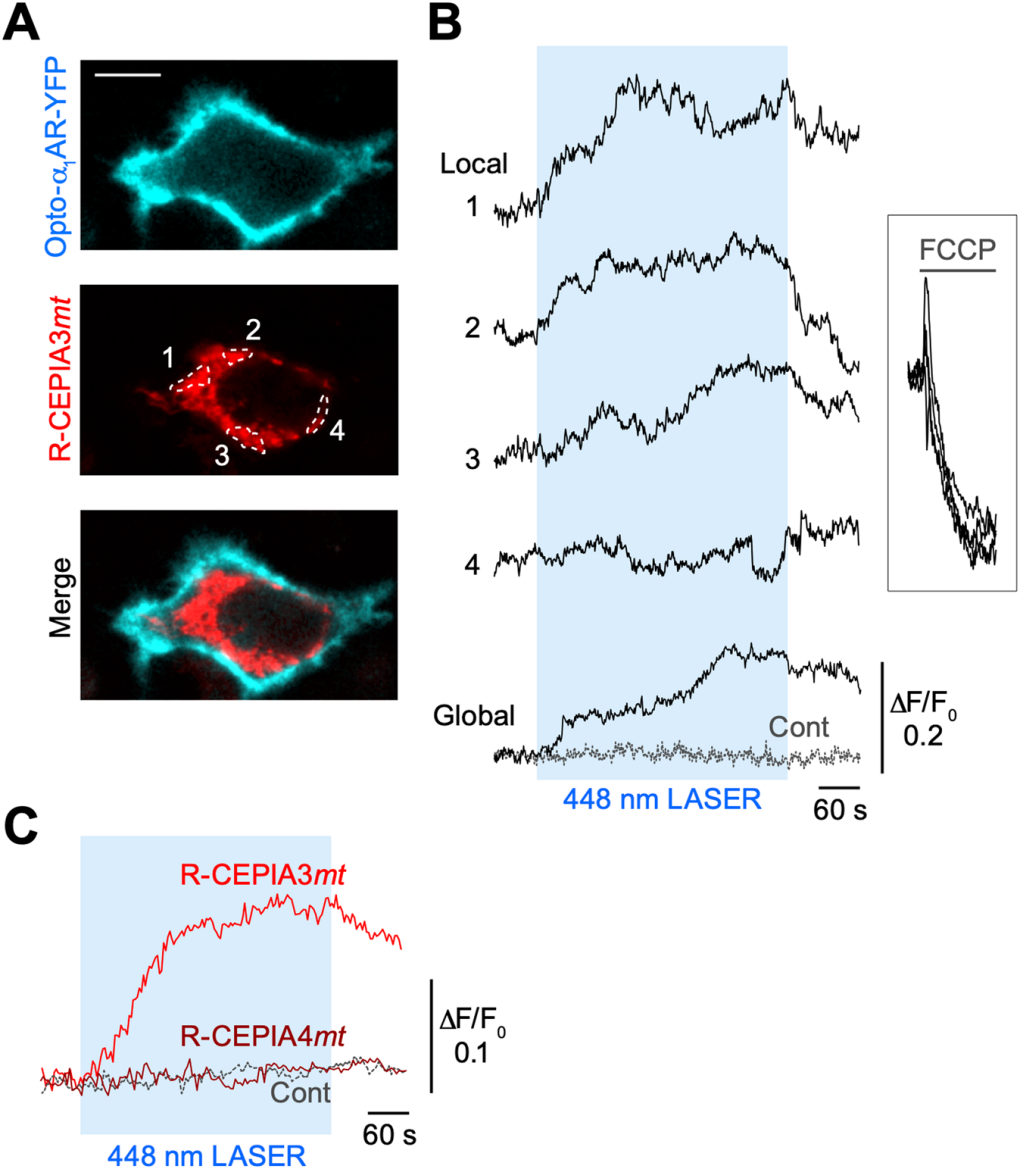
Simultaneous application of mitochondrial Ca^2+^ imaging and an optogenetic activator of the Ca^2+^ release machinery. (**A**) Representative confocal section images of Opto-α_1_AR-YFP and R-CEPIA3*mt* in a transfected HeLa cell. A merged image is shown in the lower panel. Scale bar, 10 µm. (**B**) Time courses of R-CEPIA3*mt*-reported Ca^2+^ signals in the subcellular mitochondrial domains indicated in (**A**) (*Local*; ROI 1–4). Laser irradiation (448 nm) during the period indicated by the blue box was used to activate Opto-α_1_AR-YFP, which is a chimera protein of rhodopsin, α_1_-adrenergic receptor and YFP. Traces in the right box show Ca^2+^ responses during bath application of the mitochondrial uncoupler, FCCP. Time courses of global mitochondrial Ca^2+^ signals in the same cell and in a negative control cell expressing R-CEPIA3*mt* (without expressing Opto-α_1_AR-YFP) in the same culture dish are shown at the bottom (Global and Cont). (**C**) Averaged time courses of optogenetics-induced global mitochondrial Ca^2+^ signals that were reported by R-CEPIA3*mt* (red) or R-CEPIA4*mt* (brown). As a control, the averaged response of HeLa cells transfected with only R-CEPIA3*mt* is shown (grey dashed line). *n* = 6, 3 and 3 cells for R-CEPIA3*mt*, R-CEPIA4*mt* and control, respectively, from 2 culture dishes each.

As the excitation and emission spectra of the R-CEPIA*mt* variants do not overlap with the excitation spectrum (peaking at ~450 nm) of the phospholipase C (PLC) activator, Opto-α_1_AR-YFP^22^, we performed mitochondrial Ca^2+^ imaging during optogenetic activation. In response to 448-nm laser irradiation, several mitochondrial domains (positions 1, 2 and 3 in Fig. 4A) in a HeLa cell expressing Opto-α_1_AR-YFP and R-CEPIA3*mt* showed Ca^2+^ increases (Fig. 4B), whereas another mitochondrial domain in the same cell (position 4 in Fig. 4A) did not show Ca^2+^ signals. The ensemble averaging of these local responses represents the global mitochondrial Ca^2+^ signal (bottom solid line in Fig. 4B). Another cell without Opto-α_1_AR-YFP expression in the same dish failed to show mitochondrial Ca^2+^ signals in response to light activation (“Cont” in Fig. 4B). Bath application of the mitochondrial oxidative phosphorylation uncoupler, carbonyl cyanide-p-trifluoromethoxyphenylhydrazone (FCCP), rapidly decreased the fluorescence intensity of R-CEPIA3*mt* in all mitochondrial domains, indicating that R-CEPIA3*mt* reported Ca^2+^ increases in mitochondria induced by optogenetic activation of Ca^2+^ release from the ER. Averaged traces show that the global mitochondrial Ca^2+^ responses were detected by R-CEPIA3*mt* but not by R-CEPIA4*mt* (Fig. 4C), suggesting that optogenetic PLC activation may induce mitochondrial Ca^2+^ increases up to 1–2 µM at most in our experimental conditions.

## Discussion

We succeeded to produce two variants of RFP-based mitochondrial CEPIAs, R-CEPIA3*mt* and R-CEPIA4*mt*. These new indicators have lower Ca^2+^ sensitivity than that of the previously developed high Ca^2+^ affinity RFP-based mitochondrial GECI, R-GECO1*mt* (Table 1)^15,23^. The *K*_d_ values for Ca^2+^ of R-CEPIA3*mt* and R-CEPIA4*mt* are similar to those of CEPIA3*mt* and CEPIA4*mt*, respectively, which were developed in our previous study^15^. Owing to the large dynamic range and high brightness, R-CEPIA3*mt* and R-CEPIA4*mt* allow spatiotemporal resolution imaging that is high enough to visualize heterogenous Ca^2+^ dynamics in subcellular mitochondrial domains (Fig. 3). They can be used simultaneously with other tools, such as GFP-based CEPIAs, Cal-520 and fura-2 (Fig. 3) as well as optogenetic PLC activator (Fig. 4). Furthermore, they have a lower pH dependence than the GFP-based CEPIA*mt* variants (Supplementary Fig. 2).

We have previously shown that there is no significant pH change within the mitochondrial matrix in histamine-stimulated HeLa cells^15^. Thus, the mitochondrial Ca^2+^ changes reported by R-CEPIA*mt*s in the present study are unlikely to be influenced by mitochondrial pH changes. It has been reported that under certain conditions mitochondria may undergo transient alkalizations called “mitochondrial flash” (estimated pH range: 7.5 to 8.0)^24,25^. The effect of such pH changes would be less in R-CEPIA3*mt* and R-CEPIA4*mt* than in GFP-based CEPIA*mt*s.

The mechanism underlying the intercellular and subcellular heterogeneity in mitochondrial Ca^2+^ dynamics is of great interest. Recent studies have identified molecules involved in Ca^2+^ influx through the MCU complex, including MCU, MCUb, EMRE and MICU1–3^4–11^. Furthermore, the Na^+^-Ca^2+^-Li^+^ transporter has been identified as a Ca^2+^ extrusion mechanism on the mitochondrial inner membrane^12,13^. Therefore, it is important to study whether there is intercellular and subcellular heterogeneity in the expression levels or activities of these Ca^2+^-handling proteins, and whether protein heterogeneity corresponds to the heterogeneity in mitochondrial Ca^2+^ dynamics. Moreover, it will be informative to study whether the heterogeneity in mitochondrial Ca^2+^ dynamics produces intercellular heterogeneity in cell death as well as subcellular heterogeneity in mitochondrial motility, ATP production, opening of permeability transition pores, and mitochondrial membrane potentials. Various optical tools for tagging, probing and controlling these cellular processes have been developed. Simultaneous use of these optical tools and the R-CEPIA*mt* variants is expected to make a great contribution to the field.

In summary, we added new members to the library of green and red emission mitochondrial Ca^2+^ indicators that cover a broad range of mitochondrial Ca^2+^ concentrations. Further imaging analyses using these potent and useful tools may facilitate uncovering the mode of action of mitochondrial Ca^2+^ dynamics and their functions in health and disease.

## Methods

### Gene manipulation and plasmid construction

For engineering R-CEPIA3 and R-CEPIA4, we introduced point mutations into pET19b R-GECO1^15^ using primers 1–6 (Supplementary Table 1). For mammalian expression, the cDNAs of R-CEPIA3 and R-CEPIA4 were subcloned into pCMV vector containing the mitochondria-targeting sequence (pCMV R-GECO1*mt*^15^) by restriction enzyme digestion.

### Bacterial expression and *in vitro* spectroscopy

BL21-CodonPlus(DE3)-RIL bacteria (Agilent, Santa Clara, CA, USA) were transformed with the pET19b plasmids having R-CEPIA3, R-CEPIA4, R-GECO1 or CEPIA2. The bacteria were incubated for 16–24 h at 37 °C in 2 × YT medium containing ampicillin and chloramphenicol (20 μg•ml^–1^). After harvesting the bacteria by centrifugation, the cells were resuspended in KCl/MOPS buffer (130 KCl, 50 MOPS in mM, pH 7.2) and lysed by French press (Thermo Fisher, Waltham, MA, USA) at 20,000 psi. The recombinant proteins were purified using TALON metal affinity resin (Takara Clontech, Shiga, Japan) and eluted with KCl/MOPS buffer containing 250 mM imidazole.

The absorbance spectra were measured with a NanoDrop 2000 spectrophotometer (Thermo Fisher) in KCl/MOPS buffer containing 1 mM EGTA or 1 mM CaCl_2_. The protein concentration was calculated by measuring the absorbance following alkaline denaturation, assuming ε = 38,000 M^–1^•cm^–1^ at 455 nm for R-CEPIA3, R-CEPIA4, and R-GECO1, and ε = 44,000 M^–1^•cm^–1^ at 446 nm for CEPIA2^26^. The molar extinction coefficient was calculated by dividing the peak absorbance value by the protein concentration.

Ca^2+^ titration curves were obtained by adding small aliquots of CaCl_2_ to the recombinant indicators in KCl/MOPS or KCl/HEPES (130 KCl, 50 HEPES in mM, pH 8.0) buffer. The indicators’ concentration was 25–75 nM. The Ca^2+^ concentrations were clamped with any of the following Ca^2+^ buffers: 1 mM EGTA, 1 mM BAPTA, 1 mM Br_2_BAPTA (5,5′-Dibromo BAPTA) and 1 mM Nitrilotriacetic acid (NTA). Free Ca^2+^ concentration was calculated by Maxchelator (https://somapp.ucdmc.ucdavis.edu/pharmacology/bers/maxchelator/). Fluorescence intensity was measured with F-4500 FL spectrofluorometer (Hitachi, Tokyo, Japan) at 562 ± 5/584 ± 5 nm (excitation/emission) wavelength for R-CEPIA3, R-CEPIA4, and R-GECO1, and 492 ± 10/514 ± 10 nm for CEPIA2. The obtained relationship between the Ca^2+^ concentration and the fluorescence intensity was fitted by the following single Hill plot equation with the KaleidaGraph software (Synergy Software, Reading, PA, USA).

$${F}={{F}}_{{\rm{m}}{\rm{i}}{\rm{n}}}+({{F}}_{{\rm{m}}{\rm{a}}{\rm{x}}}\,\mbox{--}\,{{F}}_{{\rm{m}}{\rm{i}}{\rm{n}}})\times {({[C{a}^{2+}]}_{{\rm{free}}})}^{{n}}/[{({[C{a}^{2+}]}_{{\rm{f}}{\rm{r}}{\rm{e}}{\rm{e}}})}^{{n}}+{({{K}{{\prime} }}_{{\rm{d}}})}^{{n}}].$$

*K*′_d_ represents apparent dissociation constant or the Ca^2+^ concentration at which half of the indicator molecules bind to Ca^2+^. *n* represents Hill coefficient. The fluorescence intensity at various Ca^2+^ concentrations was normalized by (*F* − *F*_min_)/(*F*_max_ − *F*_min_). The dynamic range of the indicator was calculated as the ratio of *F*_max_ to *F*_min_.

pH titration curves for each indicator were obtained by measuring fluorescence intensity in the solutions containing 130 mM KCl and 50 mM pH buffer (MES for pH 5.0; MES/HEPES for pH 6.0; MOPS/HEPES for pH 7.0–7.5; HEPES for pH 8.0; HEPES/CHES for pH 9.0; CHES for pH 10.0) with 1 mM EGTA or 1 mM Ca^2+^. p*K*a was obtained by fitting the obtained fluorescence intensity with a single Hill equation.

### Cell culture

HeLa cells were cultured on collagen-coated plastic dishes (IWAKI, Shizuoka, Japan) in DMEM supplemented with 10% fetal bovine serum, penicillin (100 U•ml^–1^) and streptomycin (100 U•ml^–1^). For Ca^2+^ imaging, the cells were plated on collagen type-I-coated glass-bottom dishes (MatTek, Ashland, MA, USA) and cultured for 16 h before imaging.

### Imaging

Cultured HeLa cells were transfected using Lipofectamine 3000 (Thermo Fisher) 2 or 3 days before imaging. The plasmids used in the current study were: pCMV R-CEPIA3*mt*, pCMV R-CEPIA4*mt*, pCMV CEPIA2*mt* and pcDNA3 Opto-α_1_AR-YFP. For cytosolic Ca^2+^ imaging using Cal-520 or fura-2, cells were loaded with 5 µM Cal-520 AM (AAT Bioquest, USA) or 5 µM fura-2 AM (Dojindo, Japan) at room temperature (22–24 °C) for 30 min in culture medium. Before imaging, the loading solution was washed three times and replaced with physiological salt solution (PSS) containing (in mM) 150 NaCl, 4 KCl, 2 CaCl_2_, 1 MgCl_2_, 5.6 glucose and 25 HEPES (pH 7.4).

Time-lapse images of Cal-520, R-CEPIA3*mt* and R-CEPIA4*mt* (hereafter, R-CEPIA3/4*mt*) were captured using an inverted IX81 microscope (Olympus, Tokyo, Japan) equipped with a ×20 UPlanSApo oil immersion objective [numerical aperture (NA) = 0.75; Olympus], an electron-multiplying cooled-coupled device (EM-CCD) ImagEM camera (Hamamatsu Photonics, Japan), a Lambda 10–3 filter wheel (Sutter Instrument, Navato, CA, USA), an ebx75 xenon lamp (Leica, Wetzlar, Germany) and an EL6000 metal halide lamp (Leica) at a rate of one frame per 1 or 2 s with the following set of excitation and emission filters, respectively: 472 ± 15 nm and 520 ± 17.5 nm for Cal-520; 562 ± 20 nm and 641 ± 37.5 nm for R-CEPIA3/4*mt*.

For Ca^2+^ imaging in subcellular mitochondrial domains, time lapse or snapshot images of R-CEPIA3/4*mt*, CEPIA2*mt*, fura-2 and Opto-α_1_AR-YFP were captured using a TCS SP8 confocal microscope (Leica) equipped with a × 63 HC PL APO oil immersion objective (NA = 1.40; Leica) at a rate of one frame per 1–3 s with the following wavelengths [excitation laser (nm); emission spectra (nm)]: R-CEPIA3/4*mt* (552; 560–750), CEPIA2*mt* (488; 520–550), fura-2 (405; 430–515) and Opto-α_1_AR-YFP [for confirmation of transfection by YFP fluorescence, shown in Fig. 4A (488; 500–550), for light activation (448; 530–555)]. Photobleaching was corrected for by a linear or exponential fit to the fluorescence intensity change before agonist stimulation.

For imaging of subcellular localization of CEPIA, the mitochondria in R-CEPIA3/4*mt*-expressing cells were stained by a 30-min incubation in culture medium containing 500 nM MitoTracker Green (Thermo Fisher) at 37 °C. Images were captured with a TCS SP8 confocal microscope using a ×63 HC PL APO oil immersion objective at excitation: 488 nm and emission: 500–540 nm for MitoTracker Green and excitation: 552 nm and emission: 560–750 nm for R-CEPIA3/4*mt*.

### Data analysis and statistics

Two-tailed Student’s *t*-tests were performed to determine the statistical significance using Origin 7 (OriginLab, Northampton, MA, USA). The calculations, processing and analysis of obtained images were performed with ImageJ and ImageJ Fiji (NIH, Bethesda, MD, USA). Graphs and time course traces were produced with Origin 7 and arraigned with Illustrator CC (Adobe, San Jose, CA, USA), respectively.

## Supplementary information

Sepplementary Information.
