# Supplementary material for: Red fluorescent CEPIA indicators for visualization of Ca2+ dynamics in mitochondria
Source: Sci Rep. 2020 Feb 18;10:2835. doi: 10.1038/s41598-020-59707-8 (PMC7029041; doi:10.1038/s41598-020-59707-8)
Supplement: Supplementary file 1 — Sepplementary Information. [file 41598_2020_59707_MOESM1_ESM.pdf]

Red fluorescent CEPIA indicators for visualization of Ca^{2+} dynamics in mitochondria

Kazunori Kanemaru[1#], Junji Suzuki[2#], Isamu Taiko[1,3] and Masamitsu Iino[1]*

1: Division of Cellular and Molecular Pharmacology, Nihon University School of Medicine, Tokyo 173-8610, Japan.

2: Department of Physiology, University of California San Francisco, San Francisco, CA 94158, USA.

3: Department of Physiology, Nihon University School of Medicine, Tokyo 173-8610, Japan.

#: Equal contribution

*: Correspondence should be addressed to Masamitsu Iino, Division of Cellular and Molecular Pharmacology, Nihon University School of Medicine, Tokyo 173-8610, Japan.

E-mail address: iino.masamitsu@nihon-u.ac.jp

Supplementary Figures

A

Indicator	Amino acids position in calmodulin sequence 31	36	60	63	77	92	101	111	127	133	K_d (μM) for Ca^{2+}	Dynamic range	Hill coefficient
CEPIA2 (cfGCaMP2)	E	L	N	I	K	F	S	N	E	D	0.67	5.1	2.5
CEPIA3	D	L	N	I	K	F	S	N	E	D	14.5	5.0	1.5
CEPIA4	D	L	N	I	K	W	S	N	E	E	90.2	4.9	1.7
R-GECO1	E	M	D	F	N	F	G	D	V	D	0.19	22.2	2.2
**R-CEPIA3***	D	M	D	F	N	F	G	D	V	D	3.7	30.0	1.0
**R-CEPIA4***	D	M	D	F	N	W	G	D	V	E	26.9	23.9	1.5
R-GECO1 E31D/F92W/D133E with cfGCaMP2 calmodulin#	D	L	N	I	K	W	S	N	E	E	16.6	21.9	2.3

*Developed in current study
#Reference 15

CEPIA mutations
Difference between R-GECOs and cfGCaMP2

in vitro properties at pH 7.2

B

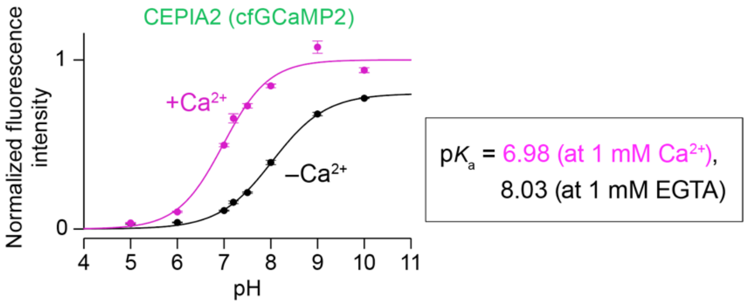

Supplementary Figure 1: Comparison of amino acids sequence of calmodulin in CEPIAs and pH titration curve of CEPIA2

A: Comparison of amino acids sequence of calmodulin in CEPIAs.

B: pH titration curve of CEPIA2 (cfGCaMP2) in Ca^{2+}-containing (1 mM, magenta) or Ca^{2+}-free (1 mM EGTA, black) solution. The plots were fitted by a single Hill equation. Mean ± SEM (n = 3).

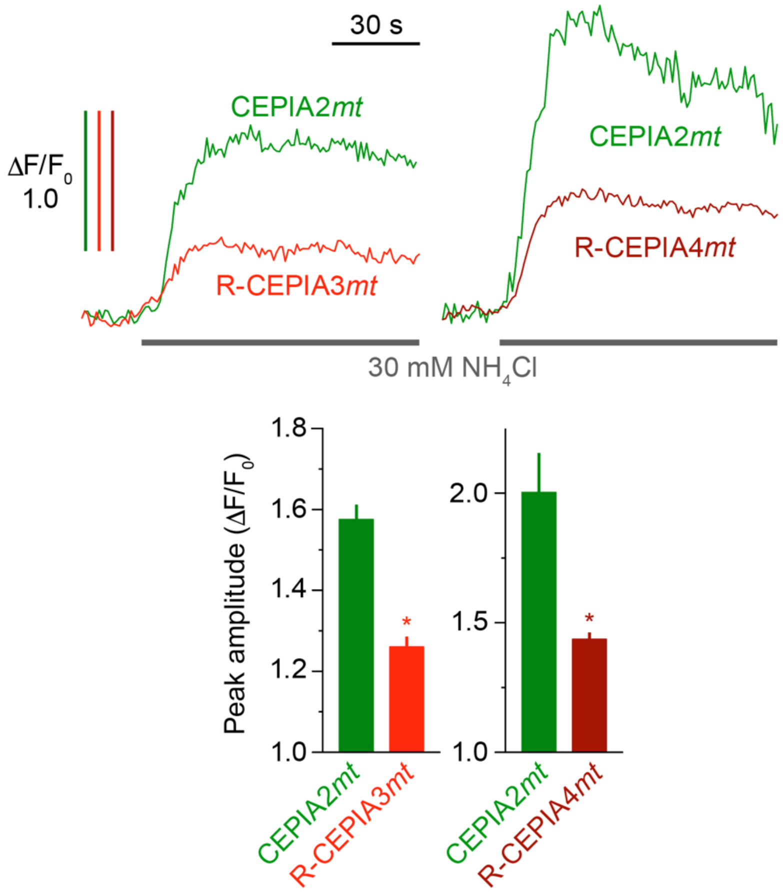

Supplementary Figure 2: Effect of alkalization on fluorescence intensity of R-CEPIA3*mt* and R-CEPIA4*mt*

Representative time courses and summarized graphs of the peak amplitude of alkalization-induced changes in the fluorescence intensity of mitochondrial CEPIAs. The data were obtained from HeLa cells expressing R-CEPIA3*mt* and CEPIA2*mt* (left) or R-CEPIA4*mt* and CEPIA2*mt* (right). n = 6 and 5 (cells), $*p = 6.64 \times 10^{-8}$ and 1.43×10^{-5}, for left and right graphs, respectively.

Supplementary Table

Supplementary Table 1: The list of primers.

Primer number		Sequence
1	(E31D)	GGGCAGAACCCCACAGAAGCAGAGCTCCAG
2	(E31D)	CAGAGACCGCATCACCGTCCCCAGATCCTT
3	(F92W)	GGGATAAGGATGGCAATGGCTACATCGGC
4	(F92W)	ACACACGGAACGCTTCGCGAATTTCCTCT
5	(D133E)	ATCGATGGAGAAGGTCAGGTAAACTACGAA
6	(D133E)	GTCTGCTACCCTGATCATTTCATCAACCTC

The primers used in the present study were listed. For the primers used for point mutations, the target mutation sites in calmodluin domain of R-GECO1 were indicated in parentheses.
